# NURSING HOME STAFFING DISPARITIES IN DISTRESSED COMMUNITIES DURING PANDEMIC

**DOI:** 10.1093/geroni/igae098.0228

**Published:** 2024-12-31

**Authors:** Yu Jin Kang, Karen Nielsen, Jacqueline Jordan, Blake McGee

**Affiliations:** Georgia State University, Atlanta, Georgia, United States; Georgia State University, Atlanta, Georgia, United States; Georgia State University, Atlanta, Georgia, United States; Georgia State University, Atlanta, Georgia, United States

## Abstract

This repeated measures study aims to examine the extent of US nursing home staffing disparities in distressed communities during the COVID-19 pandemic. The study used 2020-2022 Nursing Home Compare, 2021 LTCFocus, and the Distressed Community Index from the Economic Innovation Group. Mixed-effects models were used for data analysis. Outcomes were quarterly case-mix adjusted hours per resident day (HPRD) of all nursing staff in the last three quarters of 2020 and all of 2021: registered nurses (RN), licensed practical and vocational nurses (LP/VN), and certified nursing aides (CNA). Predictors included 2020 first quarter nurse staffing levels and distressed community index quintile. Covariates were nursing home size (greater or less than 100 beds), 5-star overall rating, Medicaid payer percentage, and non-profit status. Of the 13,845 nursing homes, 70% were for-profit and 50% had over 100 beds. On average, 5-star overall rating of nursing homes was 3.19 (sd=1.41) and Medicaid payer percentage was 60% (sd=23.5%). Compared to pre-pandemic nurse HPRD, average HPRD of the subsequent 7 quarters declined (differences: Total=-0.67, 17%; RN=-0.30, 43%; LP/VN=-0.13, 15%; CNA=-0.26, 11%). Pandemic HPRD were negatively associated with pre-pandemic HPRD (slopes: Total= -0.078; RN= -0.039; LP/VN= -0.010; CNA= -0.062, all p< 0.001). Modeled interactions suggest that declines in HPRD were greatest in the most distressed communities. Analyses showed declines in nursing home staffing levels during the pandemic, with the lowest HPRD and greatest declines observed in the most distressed communities and RNs. Practical significance of the predictors and resident outcomes requires further examination.

